# A Factor XIa Inhibitor Engineered from Banded Krait Venom Toxin: Efficacy and Safety in Rodent Models of Arterial and Venous Thrombosis

**DOI:** 10.3390/biomedicines10071679

**Published:** 2022-07-12

**Authors:** Wei Seng Chng, Aaron Wei Liang Li, Jasmine Jia Min Lim, Esther Jia En Leong, Fathiah S. Amran, R. Manjunatha Kini, Mark Yan Yee Chan, Cho Yeow Koh

**Affiliations:** 1 Department of Medicine, Yong Loo Lin School of Medicine, National University of Singapore, Singapore 117599, Singapore; weiseng@nus.edu.sg (W.S.C.); mdcalwl@nus.edu.sg (A.W.L.L.); jasmine_jm_lim@nuhs.edu.sg (J.J.M.L.); fathiahamran@gmail.com (F.S.A.); mark.chan@nus.edu.sg (M.Y.Y.C.); 2 Department of Biological Science, Faculty of Science, National University of Singapore, Singapore 117558, Singapore; estherleong5@gmail.com (E.J.E.L.); dbskinim@nus.edu.sg (R.M.K.); 3 Department of Pharmacology, Yong Loo Lin School of Medicine, National University of Singapore, Singapore 117600, Singapore

**Keywords:** factor XIa, anticoagulant, thrombosis, bleeding, venom, therapeutic, heparin, LMWH

## Abstract

Activated factor XI (FXIa) is an important antithrombotic drug target. Clinical and pre-clinical data have demonstrated that its inhibition attenuates thrombosis with minimal risk of excessive bleeding. We isolated Fasxiator from the venom of banded krait *Bungarus fasciatus* and subsequently engineered Fasxiator_N17R,L19E_, with improved affinity (K_i_ = 0.9 nM) and selectivity towards FXIa. Here, we assess the in vivo efficacy and bleeding risk of rFasxiator_N17R, L19E_ in pre-clinical animal models. Rats injected intravenously (i.v.) with bolus rFasxiator_N17R, L19E_ showed the specific in vivo attenuation of the intrinsic coagulation pathway, lasting for at least 60 min. We performed the in vivo dose-ranging experiments for rFasxiator_N17R, L19E_ as follows: FeCl_3_-induced carotid artery occlusion in rats (arterial thrombosis); inferior vena cava ligation in mice (venous thrombosis); tail bleeding time in both rats and mice (bleeding risk). Head-to-head comparisons were made using therapeutic dosages of unfractionated heparin (UFH) and low-molecular-weight heparin (LMWH) for arterial and venous thrombosis, respectively. In the arterial thrombosis model, 2 mg/kg i.v. rFasxiator_N17R,L19E_ achieved a similar antithrombotic efficacy to that of UFH, with >3-fold lower bleeding time. In the venous thrombosis model, the 10 mg/kg subcutaneous (s.c.) injection of rFasxiator_N17R,L19E_ achieved similar efficacy and bleeding levels to those of LMWH enoxaparin. Overall, rFasxiator_N17R,L19E_ represents a promising molecule for the development of FXIa-targeting anticoagulants.

## 1. Introduction

Cardiovascular and cerebrovascular diseases are the leading causes of death worldwide and account for 26% of the total deaths in 2019 [1]. Thrombosis is a major underlying pathology for these diseases, such as acute coronary syndrome (ACS), stroke, and venous thromboembolism (VTE), with the latter including deep vein thrombosis (DVT) and pulmonary embolism (PE) [2]. Anticoagulants are the major class of therapeutic agents for thrombosis treatment and prophylaxis. The goal of anticoagulation therapy is to prevent the pathological formation of blood clots during thrombosis, which can result in the occlusion of arteries and veins [3]. However, a major limitation of anticoagulant therapy is the risk of excessive bleeding [4]. An ideal anticoagulation therapy should inhibit the formation of unwanted blood clots with minimum perturbation to hemostasis by having the best balance between antithrombotic efficacy and bleeding risk (a high efficacy-to-safety index) [5].

The activated coagulation factor XI (FXIa) is a potential therapeutic target for anticoagulation with a reduced risk of excessive bleeding [4]. Contemporary views on blood coagulation hold that the amplification of coagulation occurs mainly through the intrinsic pathway. An initial amount of thrombin is generated through the tissue factor and activated factor VII (FVIIa) complex via the extrinsic pathway. Subsequently, the generated thrombin activates FXI, factor VIII, and factor V to self-amplify the coagulation signals through the intrinsic pathway [6]. While the inhibition of FXIa shuts down the intrinsic pathway, thrombin generation could still be maintained by the extrinsic pathway to a certain extent. As a result, the inhibition of FXIa tapers but does not completely abolish thrombin generation [6]. In the treatment of thrombosis, FXIa inhibition can moderate the pathological progression of coagulation and still preserve sufficient potential for hemostasis in response to vascular injury. Therefore, it is likely that FXIa inhibition can provide a favorable overall antithrombotic–hemostasis balance, which would result in minimal bleeding risk [6].

The hypothesis that FXIa is a valuable target for the development of anticoagulants with minimal risk of bleeding is strongly supported by epidemiological, animal, and clinical data [6]. One example is the observation that hemophilia C patients rarely have major bleeding complications despite having FXI deficiency [7]. Patients with severe deficiency in FXI are also found to have a reduced risk of DVT and ischemic stroke [8,9]. In addition, FXI knockout mice have reduced thrombosis but do not suffer from excessive bleeding [10,11,12,13]. Similar observations could be made in the pharmacological inhibition of FXIa in other animal models such as rats, rabbits, and baboons [14,15,16]. A few therapeutic candidates targeting FXI/FXIa have recently completed phase 2 clinical trials [4]. IONIS-FXI_RX_ (former name: ISIS-416858), an antisense oligonucleotide that targets FXI mRNA, has shown a statistically significant reduction in the incidence of VTE in patients receiving elective knee arthroplasty as compared to low-molecular-weight heparin (LMWH) enoxaparin [17]. The incidence of major or clinically relevant bleeding in an IONIS-FXI_RX_-treated group is numerically lower than enoxaparin [17]. In the FOXTROT randomized clinical trial, patients taking pre- or postoperative Osocimab (human IgG1 monoclonal antibody that inhibits FXIa) for knee arthroplasty surgery were similarly reported to have numerically lower major or clinically relevant non-major bleeding as compared to enoxaparin. Patients taking a postoperative dose (0.6 mg/kg, 1.2 mg/kg, and 1.8 mg/kg) of Osocimab were observed to have a similar incidence of VTE with enoxaparin. On the other hand, patients taking a preoperative dose of 1.8 mg/kg were found to have a statistically significant lower incidence of VTE than an enoxaparin-treated group [18]. The recent AXIOMATIC-TKR clinical trial of Milvexian (an orally available FXIa inhibitor) has also reported that the postoperative inhibition of FXIa by Milevexian could prevent venous thromboembolism in patients undergoing knee arthroplasty without major bleeding [19]. Thus, FXIa inhibitors can potentially be developed into an anticoagulant drug, with minimal bleeding complications.

The Kunitz-type protease inhibitor is an important family of serine protease inhibitors that is found in most living organisms [20]. A single Kunitz domain typically comprises of approximately 60 amino acid residues with alpha/beta folds constrained by three disulfide bonds [20]. They are functionally diverse, with activities such as the inhibition of ion channels, neutrophil elastase (anti-inflammatory), and blood coagulation factors [21,22]. Kunitz-type domains are also found in humans. For example, the tissue factor pathway inhibitor targets the extrinsic tenase complex components, (activated) factor X (FX/FXa) and FVII/FVIIa [23]. The light chain of the inter-alpha-trypsin inhibitor (bikunin) has been reported to target kallikrein (involved in endothelium permeability and airway inflammation), granzyme (involved in responses of natural killer cells and cytotoxic T-cells), and plasmin (involved in the dissolution of fibrin clots and the activation of matrix metalloproteases) [24,25,26]. The Kunitz domain of protease nexin 2 (PN2KPI) has been reported to be a potent inhibitor of FXIa, which suggests its possible role in the inhibition of FXI-dependent thrombosis in vivo [27]. The Kunitz-type domain is also a promising miniprotein scaffold being explored for the engineering of non-antibody-based protein therapeutics. Miniproteins are a diverse group of protein scaffolds (1 to 10 kDa) that have good thermal, chemical, and biological stability as well as good tissue penetration capability [28,29]. For example, ecallantide is an approved therapeutic for the treatment of hereditary angioedema and is composed of a Kunitz domain [28,29].

Previously, we reported the discovery and design of a novel FXIa inhibitor, rFasxiator_N17R,L19E_. Fasxiator is a 7 kDa Kunitz-type protease inhibitor that was isolated from the venom of a banded krait snake (*Bungarus fasciatus*) [30]. It inhibits FXIa with moderate potency (IC_50_ = 1.5 µM) while also inhibiting plasmin, FXa, and FVIIa. Through the systematic mutations of P1 and P2ʹ residues, we engineered a suitable lead for anticoagulant therapeutic development, with enhanced potency and selectivity. The variant, rFasxiator_N17R,L19E_, has >1000-fold improved inhibitory potency towards FXIa. rFasxiator_N17R,L19E_ is also at least 170-fold more selective towards FXIa as compared to other blood coagulation serine proteases. In addition, the preliminary assessment of in vivo activity by rFasxiator_N17R,L19E_ has showed some protection against thrombosis in a FeCl_3_-induced carotid artery thrombosis (CAT) model. With a fixed amount of inhibitor (0.3 mg/mice) injected through the tail vein, the occlusion time of the carotid artery was extended by approximately 3-fold. In addition to its therapeutic potential, the inhibitor has also been successfully used in a bioassay to quantify FXIa in plasma [31].

Here, we further characterize rFasxiator_N17R,L19E_ activity in vivo to understand its potential as a therapeutic candidate. First, serial blood draws were performed on rats injected with rFasxiator_N17R, L19E_ in order to obtain plasma for coagulation assays that would examine the time course of in vivo anticoagulant effects. Next, we performed dose–response experiments in rodents—namely the FeCl_3_-induced CAT model in rats and the inferior vena cava (IVC) ligation model in mice—to represent arterial and venous thrombosis, respectively. Unfractionated heparin (UFH) and LMWH enoxaparin were used in head-to-head comparisons of arterial and venous thrombosis through their respective clinically recommended dosages and routes of administration. We were able to demonstrate that rFasxiator_N17R, L19E_ can achieve a therapeutic antithrombotic level with minimal bleeding, and it is a promising molecule for the development of an FXIa-specific anticoagulant or bioassay reagent.

## 2. Materials and Methods

### 2.1. Study Approvals

All animal studies were conducted in accordance with the guidelines of the Institutional Animal Care and Use Committee (IACUC) at the National University of Singapore (NUS), under protocols R16-0008, R19-1040, and R20-0421.

### 2.2. Expression and Refolding of rFasxiator_N17R, L19E_

Briefly, a starter culture (containing a final concentration of 50 µg/mL kanamycin) of transformed SHuffle T7 *E. coli* cells (New England Biolabs, Ipswich, MA, USA) was incubated at 30 °C, with shaking at 250 rpm for 16–18 h overnight. For the expansion of the bacteria culture, 10 mL of starter culture was added to 1 L of LB broth in a 2.8 L Erlenmeyer flask. The *E. coli* was cultured at 30 °C, with shaking at 250 rpm. Expression of protein was induced using a final concentration of 0.5 mM isopropyl β-d-1-thiogalactopyranoside (IPTG) (Bio Basic, Amherst, NY, USA) when the optical density of the *E. coli* culture at 600 nm reached 0.6. After the addition of IPTG, the culture was incubated at 16 °C, with shaking at 150 rpm. After 18 h, the bacterial cell pellet was collected by centrifugation at 6000 rpm for 30 min. The pellet was resuspended in 10–15 mL of lysis buffer (1× phosphate-buffered saline (PBS), 2% Triton X-100, 5 mM dithiothreitol (DTT)) and incubated on ice, with rocking for 1 h. The resuspended pellet was sonicated for 20 min (5 s on, 5 s off) on ice and subjected to centrifugation at 10,000 rpm for 30 min at 4 °C. Inclusion bodies were obtained by collecting the pellet after the centrifugation of the cell lysate and washed (resuspend pellet with wash buffer, centrifuge at 10,000 rpm for 30 min at 4 °C, and discard supernatant) twice with 12 mL of wash buffer A (1× PBS, 2% Triton X-100, 2 M urea, 400 mM NaCI, 5 mM DTT), followed by another wash with 12 mL of wash buffer B (1× PBS, 200 mM NaCl, 5 mM DTT). For purification through a 6×-His affinity tag, the washed pellet was resuspended in 15 mL of purification buffer comprising 1× PBS, 8 M urea, 5 mM imidazole, 1 mM β-mercaptoethanol, and incubated in a column containing Ni-NTA agarose beads (Qiagen, Hilden, Germany) for 30 min. The washing and on-column refolding of rFasxiator_N17R, L19E_ were performed by the sequential flowthrough of the above purification buffer without β-mercaptoethanol and the stepwise reduction of urea concentrations from 8 M to 4 M to no urea. rFasxiator_N17R, L19E_ was subsequently eluted with an elution buffer comprising 1× PBS and 200 mM imidazole. The eluent was diluted 10× using 1× PBS and incubated with thrombin (final concentration of 125 µg/mL) overnight for the cleavage of the 6×-His tag. rFasxiator_N17R, L19E_ was further purified using reversed-phase high-pressure liquid chromatography (RP-HPLC) on a Jupiter C18 5 µm 300 Å column with a dimension of 250 × 10 mm (Phenomenex, Torrance, CA, USA). An elution gradient of 20–60% over a 10 column volume with buffer B (80% acetonitrile, 0.1% TFA) was used for the elution of rFasxiator_N17R, L19E_. The mass of the purified sample was confirmed using electrospray ionization mass spectrometry (ESI-MS).

### 2.3. Validation of FXIa Inhibition by Purified rFasxiator_N17R, L19E_

Chromogenic assay was used to determine FXIa inhibition in a buffer containing 50 mM HEPES, 140 mM NaCl, 5 mM CaCl_2_, and 0.1% BSA at pH 7.4. In a reaction well, 50 µL of human FXIa (final concentration: 0.25 nM; Prolytix, Essex Junction, VT, USA) was preincubated with 50 µL of rFasxiator_N17R, L19E_ (final concentration: 0.01, 0.03, 0.1, 0.3, 1, 3, 10, or 30 nM) for 45 min at room temperature. After the incubation, 50 µL of the chromogenic substrate S-2366 (final concentration: 1 mM; Werfen, Barcelona, Spain) was added to start the reaction. The rate of product formation was measured according to the increase in absorbance at 405 nm over 15 min. A dose–response curve was plotted using the Prism 6 software, and the IC_50_ was calculated (GraphPad, San Diego, CA, USA).

### 2.4. Animals

Rat studies were conducted on male Sprague Dawley rats weighing approximately 280–320 g. Mice studies were conducted on 8 to 9-week-old male C57BL/6NTac mice weighing approximately 25 to 30 g. Since the primary aim of the study was to establish and validate the efficacy and safety of rFasxiator_N17R,L19E_ in vivo in comparison with reference therapeutics, only animals of a single sex (male) were used to limit the potential variables. Male mice were reported to have a stronger thrombotic response [32] and were hence chosen as a more stringent challenge for rFasxiator_N17R,L19E_. Both rats and mice were obtained from InVivos (Singapore). Rats and mice were kept in microisolator cages within a facility with constant temperature, a 12 h light/dark cycle, and ad libitum access to food and water.

### 2.5. Time Course Anticoagulant Effect of rFasxiator_N17R, L19E_ in Rats

Rats were anesthetized with an intraperitoneal (i.p.) injection of ketamine/xylazine (75 mg/10 mg/kg), followed by a maintenance flow of 1% isoflurane in oxygen at 1 L/min. The femoral artery and vein were surgically exposed for blood collection and drug administration, respectively. An i.v. bolus injection (rFasxiator_N17R,L19E_ 10 mg/kg) was administered via the left femoral vein, while 300 µL blood was drawn from the left femoral artery at various time points (0, 2, 5, 10, 20, 40, 60, 90, and 120 min). The drawn blood was immediately aliquoted into individual microcentrifuge tubes containing 3.2% sodium citrate. Plasma was obtained by centrifugation at 2000× *g* for 10 min within 30 min of blood collection. The activated partial thromboplastin time (APTT) and prothrombin time (PT) were performed on the Sysmex CA-660 Coagulometer using plasma collected from rats. Actin FSL, APTT reagent (Siemens Healthcare Diagnostics, Malvern, IL, USA) and Innovin, PT reagent (Siemens Healthcare Diagnostics, IL, USA) were used in these analyses. APTT and PT were performed using default machine settings. For APTT, 50 µL of rat plasma was incubated for 1 min at 37 °C. A volume of 50 µL of Actin FSL was added to the reaction mixture and further incubated for 3 min at 37 °C. Finally, 50 µL of CaCl_2_ was added to start the reaction, and absorbance was measured at 660 nm. For PT, 50 µL of rat plasma was incubated for 3 min at 37 °C. A total of 100 µL of Innovin was added to the reaction, and absorbance was measured at 660 nm.

### 2.6. Rat Carotid Artery Thrombosis (CAT) Model

Rats were anesthetized with an i.p. injection of ketamine/xylazine (75 mg/10 mg/kg), followed by a maintenance flow of 1% isoflurane in oxygen at 1 L/min. The femoral vein was surgically exposed for drug administration. An incision was made along the midline of the neck, and the right carotid artery was surgically exposed and isolated from the surrounding tissue. A Doppler flow probe (Model: MA1PRB, Transonic System Inc., Ithaca, NY, USA) was attached to the exposed carotid artery, and blood flow was recorded using LabChart 7 Pro (ADInstruments, Colorado Springs, CO, USA). UFH, the parenteral anticoagulant of choice recommended by current clinical guidelines for the treatment of arterial thrombosis such as ACS and PCI, was used as the reference drug [33]. Saline, rFasxiator_N17R, L19E_ (0.05, 0.2, 0.5, or 2 mg/kg), or UFH (50, 100, 200, 300, or 432 U/kg) were administered via an i.v. bolus injection through a cannulation at the left femoral vein. Five minutes after the administration of rFasxiator_N17R, L19E_, thrombus formation was induced via the application of a FeCl_3_-soaked filter paper (2 × 5 mm, 10 µL of 20% FeCl_3_ solution) on the surface of the exposed carotid artery in a position distal to the flow probe for 10 min. Blood flow was measured in real time, and time-to-occlusion (TTO) due to thrombus formation was recorded when blood flow decreased to zero. The study was terminated after 60 min. TTO was recorded as 60 min if blood flow persisted throughout.

### 2.7. Rat Tail Bleeding Model

Rats were anesthetized with an intraperitoneal injection of ketamine/xylazine (75 mg/10 mg/kg), followed by a maintenance flow of 1% isoflurane in oxygen at 1 L/min. The femoral vein was surgically exposed for drug administration. Saline, rFasxiator_N17R, L19E_ (0.05, 0.2, 0.5, or 2 mg/kg), or UFH (100, 200, 300, or 432U/kg) were administered via an i.v. bolus injection through a cannulation at the left femoral vein. Five minutes after the administration of rFasxiator_N17R, L19E_, a spring-loaded blade device (Surgicutt Adult bleeding time device; ITC, Piscataway, NJ, USA) was used to make a longitudinal incision (1 mm depth × 5 mm length) on the ventral side of the rat’s tail (9–9.5 cm from the tip of the tail). Blood from the site of incision was blot dried (from the side of the wound, without touching the wound) with a filter paper every 15 s until the bleeding ceased. Tail bleeding time (TBT) was defined as the time after incision until the cessation of bleeding is observed within eight consecutive blotting periods. The study was terminated 60 min after tail incision. TBT was recorded as 60 min if the bleeding persisted throughout.

### 2.8. Mouse Inferior Vena Cava (IVC) Ligation Model

Mice were anesthetized with an intraperitoneal injection of ketamine/xylazine (75 mg/10 mg/kg), followed by a maintenance flow of 1% isoflurane in oxygen at 1 L/min. An incision was made with surgical scissors along the abdominal midline, 1.5 cm down from the sternum. The abdomen was then accessed through the linea alba, and the intestines were exteriorized to the mouse’s left with the use of sterile cotton buds moistened with sterile warm (37 °C) saline. The intestines were then covered with sterile gauze soaked in warm (37 °C) saline to prevent them from drying out. The IVC was then identified, and with forceps, a careful diverging movement was applied to create a hole between the aorta and the IVC. A 7-0 inert Prolene suture was carefully threaded through the hole with forceps, and a loose knot was tied around the IVC, just below the left renal vein. The loose knot was then tightened to ligate the IVC, and another knot was tied to secure the ligation. All visible side branches connected to the IVC were also ligated. After ligation, the intestines were carefully pushed back into the peritoneal cavity and distributed equally. The incision was then sutured in 2 layers. The peritoneum was closed with 6-0 vicryl sutures using continuous loops, with both ends secured with knots. The skin was closed with 6-0 monofilament sutures through interrupted loops. Iodine was applied over the stitches to minimize the risk of infection. Anesthetic reversal (atipamezole, 0.1 mL/10 g) was administered through i.p. injection to speed up recovery. LMWH enoxaparin was chosen as the reference drug because the parenteral anticoagulant is recommended as the initial or lead-in treatment for acute DVT and PE by current clinical guidelines [34,35]. Saline, rFasxiator_N17R__,L19E_ (5 mg/kg or 10 mg/kg), or enoxaparin (18.5 mg/kg), together with 0.5 mL of warm (37 °C) saline (for hydration) and buprenophrine (0.1 mg/kg, for analgesic treatment), were injected subcutaneously (s.c.) before placing the mice (with heat pad) back into their cage to recover. Twenty-four hours after the surgery, the mice were euthanized after the tail bleeding experiment (see Section 2.9), and their IVCs were excised. The full length of the thrombi formed in the IVCs were extracted, dried for 30 min, and weighed.

### 2.9. Mouse Tail Bleeding Model

Mice that had undergone IVC ligation and had been injected s.c. with saline, rFasxiator_N17R, L19E_ (5 mg/kg or 10 mg/kg), or enoxaparin (18.5 mg/kg) 24 h prior were subjected to tail bleeding experiments before euthanization. The mice were anesthetized with an i.p. injection of ketamine/xylazine (75 mg/10 mg/kg), followed by a maintenance flow of 1% isoflurane in oxygen at 1 L/min. The mice were placed in prone position on a 37 °C heating plate, with their tails hanging off the edge of the heating plate. Their tails were then soaked in warm (37 °C) saline for 5 min. After soaking, the mice tails were first dried with paper towels before slotting their tails through a snipped-off 10 uL pipette tip. A scalpel blade was used to transect the tail that had passed through the pipette tip. The transacted tail was then immediately placed into a new warm (37 °C) saline for a maximum period of 5 min. TBT was observed. The collected blood was lysed using the freeze–thawing method, and its hemoglobin content was analyzed according to the protocol of the Hemoglobin Assay Kit (Sigma-Aldrich, St. Louis, MO, USA).

### 2.10. Statistical Analysis

All statistical analyses and curve-fitting by non-linear regression were performed using Prism 6 (GraphPad, CA, USA). The dose-response curve to determine the IC_50_ of rFasxiator_N17R, L19E_ was used and ascertained with goodness-of-fit analyses, as implemented by Prism 6. The statistical difference between clotting times for saline and rFasxiator_N17R,L19E_ for the analysis of time course anticoagulant effect in rats, was analyzed by two-way analysis of variance (ANOVA), followed by Fisher’s Least Significant Difference (LSD) test for significance at each of the time points. Statistical difference among the saline and treatment groups in CAT model was analyzed by one-way ANOVA, followed by Tukey’s multiple comparison test. All data are expressed as mean ± standard error of mean (SEM), unless stated otherwise.

## 3. Results

### 3.1. Recombinantly Expressed rFasxiator_N17R,L19E_ Inhibits FXIa Amidolytic Activity

**Figure 1:**
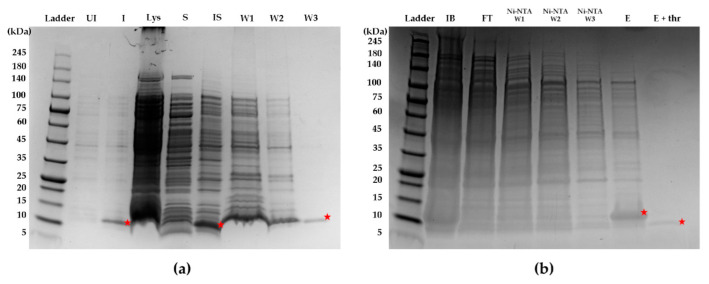
Sodium dodecyl sulfate-polyacrylamide gel electrophoresis (SDS-PAGE) of the process of rFasxiator_N17R, L19E_ expression and purification. (**a**) Lane 1: protein ladder (Ladder), Lane 2: bacteria pellet before IPTG induction (UI), Lane 3: bacteria pellet after IPTG induction (I), Lane 4: cell lysate (Lys), Lane 5: soluble fraction (S), Lane 6: insoluble fraction (IS), Lane 7: supernatant after step 1 washing of IS (W1), Lane 8: supernatant after step 2 washing of IS (W2), Lane 9: supernatant after step 3 washing of IS (W3); (**b**) Lane 1: protein ladder (Ladder), Lane 2: inclusion bodies resuspended in purification buffer (IB), Lane 3: flowthrough during sample application on Ni-NTA column (FT), Lane 4: wash 1 (Ni-NTA W1), Lane 5: wash 2 (Ni-NTA W2), Lane 6: wash 3 (Ni-NTA W3), Lane 7: elution (E), Lane 8: eluent after overnight incubation with thrombin (E + thr). Bands representing rFasxiator_N17R,L19E_ throughout the purification process are marked with a red star. The expected mass of the uncleaved his-tag-rFasxiator_N17R,L19E_ is approximately 12 kDa. The expected mass of rFasxiator_N17R, L19E_ after thrombin cleavage of 6×-His tag is approximately 7 kDa.

**Figure 2:**
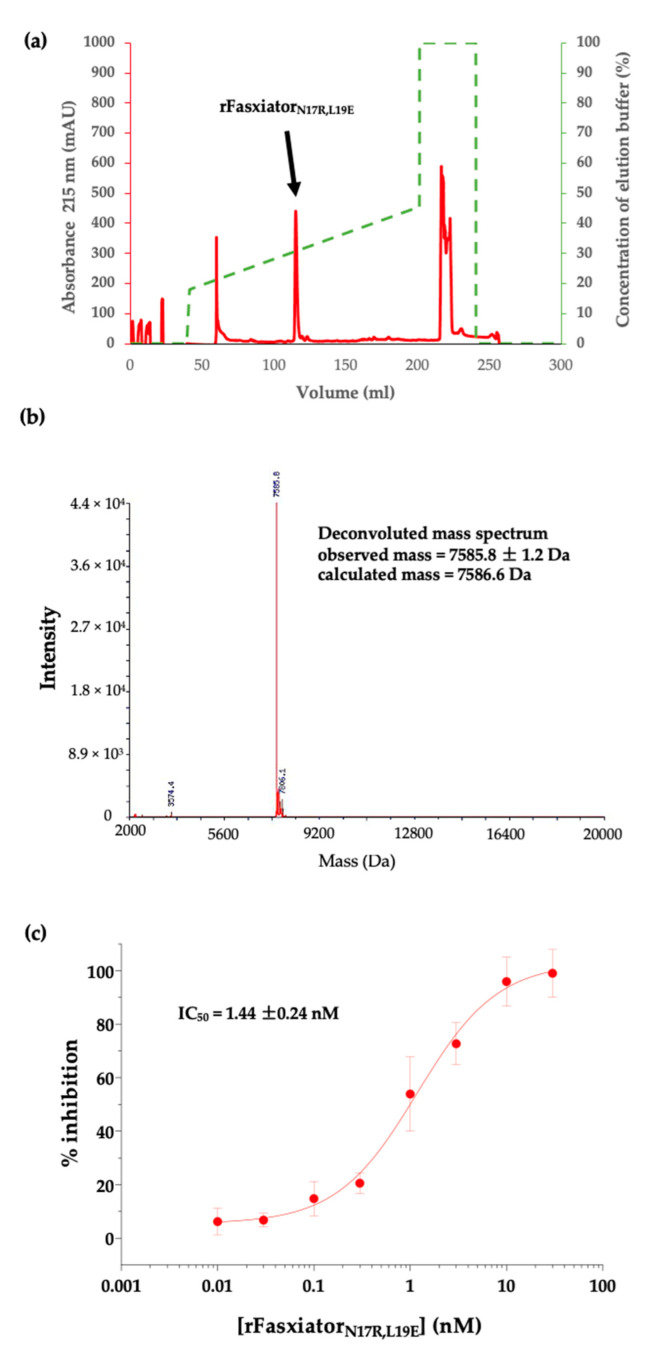
Purification and validation of FXIa inhibition by rFasxiator_N17R, L19E._ (**a**) RP-HPLC chromatogram of rFasxiator_N17R, L19E_. The arrowed peak indicates the elution of rFasxiator_N17R, L19E_. (**b**) ESI-MS deconvoluted spectrum of purified rFasxiator_N17R, L19E_ with an observed mass of 7585.8 Da, consistent with the mass of the protein calculated from the amino acid sequence. (**c**) Dose–response curve of FXIa inhibition by rFasxiator_N17R, L19E_ with IC_50_ = 1.44 ± 0.24 nM (*n* = 5).

rFasxiator_N17R, L19E_ was overexpressed as an insoluble protein in the inclusion bodies (Figure 1) despite the use of SHuffle T7 *E. coli* cells, which constitutively express the disulfide bond isomerase in order to promote the formation of disulfide bonds with correctly paired cysteines. Therefore, we optimized our earlier protocol for the purification and on-column refolding of rFasxiator_N17R, L19E_ from inclusion bodies [30]. For an inclusion body pellet obtained from a 1L culture, the sample yield after Ni-NTA elution was low, at approximately 0.95 mg. The 6× -His tag was successfully cleaved from the expressed rFasxiator_N17R, L19E_ using thrombin after elution from the Ni-NTA column, as shown in Figure 1b. rFasxiator_N17R, L19E_ was further purified to homogeneity using RP-HPLC after affinity purification and his tag cleavage, eluting at approximately 30% buffer B (Figure 2a). Our RP-HPLC and ESI-MS data show the successful purification of rFasxiator_N17R, L19E_, with the expected mass of 7585.8 kDa (Figure 2b). The amidolytic activity of FXIa on the chromogenic substrate S-2366 was inhibited by rFasxiator_N17R, L19E_ in a dose-dependent manner. The IC_50_ calculated from the dose–response curve is 1.44 ± 0.24 nM (Figure 2c), which is similar to the previously reported value of 1.26 nM [30].

### 3.2. rFasxiator_N17R,L19E_ Prolonged APTT in Rats for at Least 60 Min

**Figure 3:**
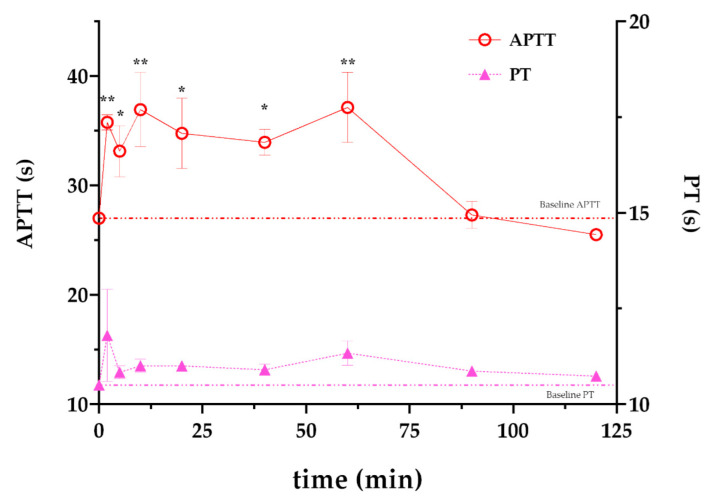
Time course anticoagulant effect of rFasxiator_N17R, L19E_ in rats. Activated partial thromboplastin time (APTT, red open circles, left *y*-axis) and prothrombin time (PT, pink solid triangles, right *y*-axis) of rat plasma collected at 2, 5, 10, 20, 40, 60, 90, and 120 min after a single i.v. bolus injection of rFasxiator_N17R, L19E_ (*n* = 3). Baseline APTT and PT (time = 0 min) are plotted as red and pink dotted lines, respectively. ** indicates that *p* ≤ 0.01; * indicates that *p* ≤ 0.05.

As an approximation of the plasma half-life of rFasxiator_N17R, L19E_ in vivo, we investigated the duration of the anticoagulant action of rFasxiator_N17R, L19E_ in rats after a single i.v. bolus injection. rFasxiator_N17R, L19E_ was administered through the femoral vein, and blood was collected at fixed intervals over a duration of 2 h. The plasma collected was used for the APTT and PT assays to assess the effect on the intrinsic and extrinsic coagulation pathways, respectively. Our data showed that APTT was maintained above baseline for at least 60 min after the injection of rFasxiator_N17R, L19E_ before returning to baseline when sampled at 90 min (Figure 3). PT remained similar and close to the baseline throughout the 120 min.

### 3.3. Carotid Artery Thrombosis (CAT) and Tail Bleeding Model in Rats

**Figure 4:**
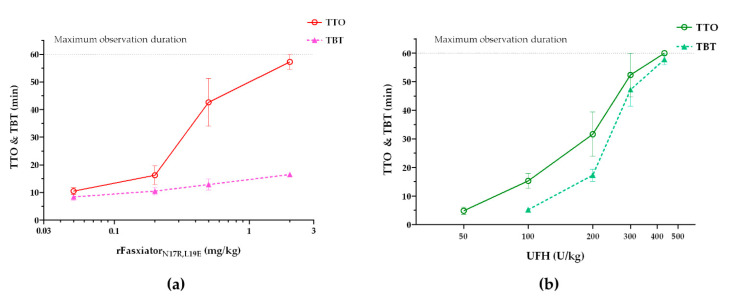
FeCl_3_-induced carotid artery thrombosis (CAT) and tail bleeding model in rats. (**a**) Time-to-occlusion (TTO) of the FeCl_3_-induced CAT model in rats treated with rFasxiator_N17R, L19E_ at 0.05 (*n* = 6), 0.2 (*n* = 6), 0.5 (*n* = 6), and 2 (*n* = 4) mg/kg were plotted as red open circles. Tail bleeding time (TBT) of rats treated with rFasxiator_N17R, L19E_ at 0.05 (*n* = 6), 0.2 (*n* = 6), 0.5 (*n* = 6), and 2 (*n* = 6) mg/kg were plotted as pink solid triangles. The gray dash line represents the maximum observation duration of 60 min in both models. (**b**) TTO of the FeCl_3_-induced CAT model in rats treated with unfractionated heparin (UFH) at 50 (*n* = 5), 100 (*n* = 6), 200 (*n* = 7), 300 (*n* = 5), and 432 (*n* = 5) U/kg were plotted as dark green open circles. TBT of rats treated with UFH at 100 (*n* = 6), 200 (*n* = 7), 300 (*n* = 5), and 432 (*n* = 5) U/kg were plotted as light green solid triangles. The gray dotted line represents the maximum observation duration of 60 min in both models.

In the arterial thrombosis model, 20% FeCl_3_ was used to induce CAT, and rFasxiator_N17R, L19E_ was administrated via i.v. through the cannulation at the right femoral vein to ensure the complete delivery of the molecule. Dose–response experiments were performed in comparison with UFH, the recommended parenteral anticoagulant for clinical use in ACS and percutaneous coronary intervention (PCI) [33]. TTO in rats treated with saline was 8.0 ± 2.1 min. TTO in rats treated with rFasxiator_N17R,L19E_ and UFH increased with increasing dosage, demonstrating the dose-dependent efficacy of both molecules in the disease model (Figure 4). The clinically recommended dosage of UFH for PCI is 70 U/kg (i.v.), and it is translated to its animal equivalent dose of 432 U/kg for rats [36]. At this therapeutic dose, UFH achieved maximum antithrombotic efficacy in which no occlusion of the carotid artery was observed during the whole duration of observation (60 min) (Figure 4b). rFasxiator_N17R, L19E_ achieved the same maximum antithrombotic efficacy (i.e., equivalent to the therapeutic dose of UFH) at 2 mg/kg. (Figure 4a). In separate groups of animals treated with saline, rFasxiator_N17R, L19E_, and UFH, the bleeding time after an incision was made on the tail vein also increased with increasing dosages of the respective molecules, demonstrating dose-dependent bleeding risks. TBT in rats treated with saline was 7.5 ± 0.67 min. However, the increase in TBT in rats treated with rFasxiator_N17R,L19E_ was demonstrably lower than those treated with UFH. At their respective therapeutic doses, TBT in rats treated with 2 mg/kg rFasxiator_N17R,L19E_ was 16.5 ± 1.35 min, approximately 3.5 times lower than TBT in rats treated with 432 U/kg of UFH (57.8 ± 4.4 min), suggesting a wider efficacy-to-safety index for rFasxiator_N17R,L19E_ than that for UFH (Figure 4).

### 3.4. Inferior Vena Cava (IVC) Ligation and Tail Bleeding Model in Mice

**Figure 5:**
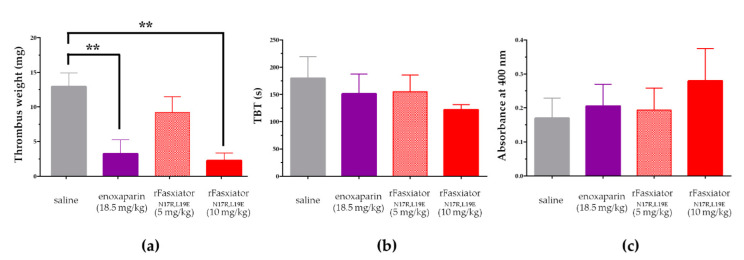
Inferior vena cava (IVC) ligation and tail bleeding model in mice. (**a**) Weight of thrombus from the inferior vena cava ligation of mice treated with saline (gray, *n* = 8), enoxaparin (purple, *n* = 8), 5 mg/kg rFasxiator_N17R, L19E_ (red checked, *n* = 8), and 10 mg/kg rFasxiator_N17R, L19E_ (solid red, *n* = 9). ** indicates that *p* ≤ 0.01 in Tukey’s multiple comparison test. (**b**) Bleeding time following transection of mice tail (TBT) treated with saline (gray, *n* = 6), enoxaparin (purple, *n* = 8), 5 mg/kg rFasxiator_N17R, L19E_ (checkered red, *n* = 6), and 10 mg/kg rFasxiator_N17R, L19E_ (solid red, *n* = 5). (**c**) Hemoglobin assay of blood collected from the transected tail of mice that were treated with saline (gray, *n* = 6), enoxaparin (purple, *n* = 8), 5 mg/kg rFasxiator_N17R, L19E_ (red checkered, *n* = 6), and 10 mg/kg rFasxiator_N17R, L19E_ (solid red, *n* = 5) 24 h before the procedure.

We performed IVC ligation experiments in a mouse DVT model [37]. rFasxiator_N17R, L19E_ was compared to LMWH enoxaparin, the recommended anticoagulant for clinical use in acute DVT. The clinically recommended dose of enoxaparin for the treatment of DVT is 1.5 mg/kg once daily s.c. or 1 mg/kg s.c. every 12 h [34,35]. We chose the once daily dose, which translates to an equivalent dose of 18.5 mg/kg in mice for the experiments [36]. In respective groups of mice, s.c. injections of saline, 5 mg/kg rFasxiator_N17R, L19E_, 10 mg/kg rFasxiator_N17R, L19E_, and 18.5 mg/kg enoxaparin were administrated within 15 min after the ligation of the IVC. The IVC were isolated after 24h to measure the weight of the thrombus formed. The thrombus weight in 10 mg/kg rFasxiator_N17R, L19E_-treated mice (2.2 ± 1.1 mg) and enoxaparin-treated mice (3.2 ± 2.0 mg) showed a statistically significant reduction (** *p* ≤ 0.01) in thrombus size as compared to the saline-treated mice (13 ± 2.0 mg) (Figure 5a). The thrombus weight of mice that were treated with 5 mg/kg rFasxiator_N17R, L19E_ (9.2 ± 2.3 mg) is numerically lower than that of the saline-treated mice, although the difference is not statistically significant (Figure 5a). To assess the effect of rFasxiator_N17R, L19E_ on bleeding in mice, tails were transected before the procedure to isolate the IVC (24 h after receiving respective drug treatments), and the bleeding time was recorded. The blood was collected and used to measure the hemoglobin level as an approximation to the volume of blood loss. No statistical difference was found between the doses of rFasxiator_N17R, L19E_, the enoxaparin-treated group, and the saline-treated group for both TBT and absorbance in the hemoglobin assay (Figure 5b,c). Although we did not detect statistical significance in the hemoglobin level across all treatment groups, we observed that TBT (Figure 5b) trended directly opposite of the hemoglobin level for all groups, resulting in the lack of correlation between TBT and blood loss. However, considering the low absolute absorbance values from the readings of the hemoglobin assays (Figure 5c) and the overall lack of statistical significance, it may be premature to draw any further conclusions without additional investigations.

## 4. Discussion

The assessment of in vivo efficacy in disease-relevant animal models of molecules of interest in comparison to appropriate reference treatments is a crucial step in the development of any therapeutic. For anticoagulants, bleeding tendency can also be studied in animals as a safety indicator. In the present study, we evaluated rFasxiator_N17R,L19E_ for these purposes. We expressed and refolded rFasxiator_N17R, L19E_ to its functional conformation, with an IC_50_ similar to a previously reported value [30]. We observed that rFasxiator_N17R, L19E_ inhibits the intrinsic pathway (prolongation of APTT) in rats for at least 60 min, while the extrinsic pathway remains unmodified (no considerable change in PT over time). In our CAT model, for the assessment of arterial thrombosis, we showed that 2 mg/kg of rFasxiator_N17R, L19E_ i.v. achieved a similar antithrombotic efficacy (60 min occlusion time) to that of UFH at the clinically recommended dose for PCI, with 3.5× lower bleeding time. In our IVC ligation model, for the assessment of venous thrombosis, we showed that 10 mg/kg of rFasxiator_N17R, L19E_ s.c. achieved a similar antithrombotic efficacy to that of the therapeutic dose of enoxaparin without increasing baseline bleeding time at 24 h. Overall, we have shown that our FXIa-inhibiting miniprotein rFasxiator_N17R, L19E_ can achieve a similar efficacy to that of reference therapeutics in both arterial and venous thrombosis models.

There is considerable interest in developing the Kunitz domain as miniprotein therapeutics [28,29]. Several exogenous inhibitors of FXIa with the Kunitz domain were identified from venomous animals or the saliva of hematophagous animals. In addition to rFasxiator_N17R, L19E_, other examples include Ir-CPI from the castor bean tick (*Ixodes ricinus*), Desmolaris from the vampire bat (*Desmodus rotundus*), WPK5 (and its mutant WPK5-Mut) from the leech (*Whitmania pigra*), and DAKS1 from the sharp-nosed viper (*Deinagkistrodon acutus*) [38,39,40,41]. Each of these inhibitors have their own unique characteristics. All molecules generally showed good antithrombotic efficacy with low bleeding risk in animal models [38,39,40,41]. However, the selectivity profiles of these inhibitors against different coagulation and fibrinolytic serine proteases vary. Among all coagulation and fibrinolytic enzymes, rFasxiator_N17R, L19E_ is most selective towards FXIa (K_i_ = 0.86 nM), while the K_i_(s) against other coagulation enzymes is at least 170-fold higher (next low K_i_ is against plasmin at 146 nM) [30]. In contrast, Ir-CPI binds to FXIa, FXIIa, and plasmin with similar affinities at the nanomolar level, and to kallikrein at the micromolar level [40]. Similarly, Desmolaris binds to FXIa and FXa with similar K_i_(s) of 12 and 15 nM, while inhibiting kallikrein with a slightly weaker activity [39]. WPK5-Mut and DAKS1 also appeared to be FXIa-selective by at least 15- and 24-fold against other coagulation and fibrinolytic enzymes (86% inhibition when the WPK5-Mut:FXIa ratio was at 50:1, 28% inhibition when the WPK5-Mut:FXa ratio was at 775:1; 70% inhibition when the DAKS1:FXIa ratio was at 125:1, 62% inhibition when the DAKS1:Kallikrein ratio was at 3000:1) [38,41]. Although we did not perform head-to-head comparisons of rFasxiator_N17R, L19E_ and the other exogenous FXIa inhibitors, the in vivo activity of rFasxiator_N17R, L19E_ was consistent with these molecules. For example, in the CAT model, 2 mg/kg rFasxiator_N17R, L19E_ (i.v.) showed maximum antithrombotic efficacy, similar to the therapeutic dose of UFH. In comparison, it was reported that 3 mg/kg of WPK5-Mut (i.v.) or 2.6 mg/kg of DAKS1 showed similar antithrombotic efficacy compared to UFH as positive control [38,41]. Other than rFasxiator_N17R,L19E_, Ir-CPI was the only other molecule tested in a venous thrombosis model. Although Ir-CPI at 1 mg/kg appeared to reduced thrombus formation in the IVC ligation model, major differences in the experiments made the comparison with rFasxiator_N17R, L19E_ challenging. For example, rFasxiator_N17R, L19E_ was injected s.c. to allow for direct comparison with the reference therapeutic enoxaparin. In contrast, Ir-CPI was injected i.v., and the experiments did not include a reference therapeutic such as enoxaparin. Nevertheless, the discovery of different exogenous inhibitors continues to enrich the development pipeline of molecules targeting FXIa, an important antithrombotic drug target.

Despite the availability of several anticoagulant therapeutics, rFasxiator_N17R,L19E_ as a miniprotein-based, potent, and specific FXIa inhibitor has a niche as a parenteral, short-term (a course that lasts between an hour and a few days) anticoagulant for acute arterial and venous arterial thrombosis. For prophylaxis and treatment against acute arterial thrombosis such as ACS and PCI, parenteral injectable anticoagulants with a fast onset and a short duration of action are preferable since the antithrombotic effect can be easily adjustable on demand [42]. For example, during PCI, UFH is given as a bolus i.v. injection of 70–100 U/kg (with top-ups, when necessary) [33] and has a duration of action of up to 4 h [43]. Bivalirudin, an FDA-approved thrombin inhibitor indicated for PCI, is usually given as a bolus i.v. injection of 0.75 mg/kg, followed by a 1.75 mg/kg/h continuous i.v. infusion, with a short half-life of 25 min [44]. Our study suggests that rFasxiator_N17R, L19E_ is likely to have a slightly longer duration of action (between 60 min and 90 min) when administered i.v., which is consistent with the short half-life of other low-molecular-weight proteins and peptides [45]. However, rFasxiator_N17R, L19E_ may be advantageous for indications such as PCI, whereby a single i.v. bolus will generate enough antithrombotic efficacy for peri-procedural protection against stent thrombosis, with a reduced risk of post-procedural bleeding due to fast clearance (typical PCI procedure lasts for 30–60 min). Furthermore, rFasxiator_N17R, L19E_ as an FXIa-targeting inhibitor is likely to have a low inherent bleeding risk. In contrast, the use of UFH comes with many challenges and health risks. Apart from the risk of bleeding, the unpredictable and variable pharmacokinetic profile of UFH makes it challenging to provide an optimal dose for treatment [46]. It is reported that the failure to achieve the appropriate therapeutic concentration of UFH during the early treatment of an acute thrombotic event is associated with a several-fold increase in the recurrence of DVT and myocardial infarction (MI) in patients [46]. UFH can also cause serious drawbacks such as heparin-induced thrombocytopenia, osteoporosis, and hypersensitivity reaction [4]. On the other hand, bivalirudin is expensive and may not offer obvious clinical advantages over UFH in the management of patients undergoing PCI [47]. Moreover, a meta-analysis study has reported that the use of bivalirudin has a higher risk of MI and stent thrombosis as compared to UFH [48]. Parenteral FXIa inhibitors under clinical development may also not be suitable for the treatment of acute thrombosis. IONIS-FXI_RX_ has slow onset and offset of action [3]. The maximum reduction of FXIa using IONIS-FXI_RX_ would take 3–4 weeks of treatment, and the restoration of FXIa back to its baseline level would take several weeks after stopping treatment [3]. Although Osocimab has a fast onset of activity after its infusion [49], it has a long half-life of 30 to 40 days, which would take several weeks to be completely eliminated from the body after treatment [18]. The orally available Milvexian only reached steady-state plasma concentration within 3 to 6 days [50]. Therefore, rFasxiator_N17R, L19E_ remained the good therapeutic candidate for the development of a parenteral anticoagulant against acute thrombosis diseases.

Similarly, for the inpatient treatment of acute VTE, parenteral, fast-onset anticoagulants remain crucial. Short-term (days), repeated s.c. dosing of LMWH is usually used at the initiation of anticoagulant treatments for DVT and PE [34,35]. Therefore, we tested rFasxiator_N17R, L19E_ in comparison with LMWH enoxaparin in the IVC ligation model in mice. Despite the apparent short duration of action for rFasxiator_N17R, L19E_ when injected i.v. (Figure 3), the s.c. injection of rFasxiator_N17R, L19E_, albeit at the higher dose of 10 mg/kg, allowed for the effective prevention of clot formation in the ligated IVC, which was sustained throughout the 24 h experimental period. This shows that rFasxiator_N17R, L19E_ may also be a viable option as a parenteral anticoagulant for the treatment of acute venous thromboembolism.

Our study is not without limitations. First, the bacterial expression of rFasxiator_N17R, L19E_ resulted in an insoluble protein and a low overall yield, posing as scale-up and manufacturing challenge for therapeutic development. An optimized recombinant expression process in mammalian or yeast cells would be needed in the future. Second, due to the fast clearance of rFasxiator_N17R,L19E_, we had to use a high concentration of rFasxiator_N17R,L19E_ in addition to the s.c. injection in the IVC ligation model to demonstrate its sustainable efficacy over a 24 h period. However, appropriate modifications of the molecule such as conjugation with polyethylene glycol, fatty acid chain, and fusion with IgG Fc may drastically prolong the half-life of the molecule [51]. Finally, animal in vivo data may not be directly applicable to humans. Further studies should be performed on human samples to validate the effect of rFasxiator_N17R, L19E_ for human use.

## 5. Conclusions

In summary, our study highlights that rFasxiator_N17R, L19E_ is as effective as conventional therapeutics, such as UFH and LMWH, for the treatment of acute arterial and venous thrombosis and has a reduced bleeding risk. There are currently three FXIa inhibitors that have completed Phase II clinical trials, but the need for a fast-onset, short-acting (hours to a day), parenteral FXIa inhibitor to complement or compete with UFH and LMWH remains. We have demonstrated that rFasxiator_N17R,L19E_ is a suitable candidate to meet this need. In addition, the demonstration of the in vivo activity of rFasxiator_N17R,L19E_ here also further strengthens the basis for its development as an FXIa-targeting bioassay reagent in order to quantify FXIa in plasma or buffer, as reported elsewhere [31].
